# Experimental Airborne Transmission of Porcine Postweaning Multisystemic Wasting Syndrome

**DOI:** 10.1155/2013/534342

**Published:** 2013-02-07

**Authors:** C. S. Kristensen, C. K. Hjulsager, K. Vestergaard, K. Dupont, V. Bille-Hansen, C. Enøe, S. E. Jorsal, P. Bækbo, L. E. Larsen

**Affiliations:** ^1^Pig Research Centre, Danish Agriculture & Food Council, Vinkelvej 11, 8620 Kjellerup, Denmark; ^2^National Veterinary Institute, Technical University of Denmark, Bülowsvej 27, 1870 Frederiksberg C, Denmark

## Abstract

The objective of these studies was to investigate if porcine postweaning multisystemic wasting syndrome (PMWS) could be induced in healthy pigs following contact with air from pigs with clinical signs of PMWS. The pigs were housed in different units. Either 31 (study I) or 25 (study II) pigs with clinical symptoms of PMWS from a PMWS-affected herd and 25 healthy pigs from a PMWS-free, but PCV2-positive, herd were housed in unit A. Fifty pigs from a PMWS-free herd were housed in unit B, which were connected by pipes to unit A. In unit C, 30 pigs from a PMWS-free herd were housed as controls. In study II, the pigs in units A and B from the PMWS-free herd developed clinical signs of PMWS 2-3 weeks after arrival. PMWS was confirmed at necropsy and the diseased pigs had increased PCV2 load and increased antibody titers against PCV2 in serum that coincided with the development of clinical signs typical of PMWS. Sequence analysis revealed that the PCV2 isolate belonged to genotype 2b. In conclusion, the present study showed that PMWS can be induced in pigs from a PMWS-free herd by airborne contact with pigs from a PMWS-affected herd.

## 1. Introduction 

Postweaning multisystemic wasting syndrome (PMWS) is an important disease in weaned pigs worldwide. PMWS was first described in Canada in 1991 as a chronic disease with progressive weight loss in pigs from 4–16 weeks of age [1]. Since then, the disease has been diagnosed in many countries in North America, Asia, and Europe including Denmark [2, 3]. The clinical signs of PMWS comprise unthriftiness/wasting, paleness of the skin, enlarged lymph nodes, and occasionally jaundice, respiratory symptoms, or diarrhoea [1, 3, 4]. Affected pigs have lesions in lymphoid organs characterized by lymphoid depletion and the presence of giant cells and inclusion bodies [4–7]. PCV2 has proved to be necessary but not sufficient for development of PMWS, since the virus is present in both affected and PMWS-free pigs and herds [4, 8].

The PCV2 virus is transmitted between pigs by the oro-fecal and/or respiratory routes [9, 10] and vertical transmission has also been documented [4, 11]. The high prevalence of PCV2 in almost all herds of all pig-producing countries indicates that the transmission of PCV2 is very effective [12–15]. In contrast, only a few studies have been performed on the “transmission” of the PCV2-associated disease complexes (PCVDs), that is, whether PMWS can be “transmitted” from PMWS-affected to PMWS-free pigs. A study performed in New Zealand demonstrated disease development in healthy pigs in direct or indirect contact with PMWS-affected pigs when they were mingled at 4 weeks of age but not when they were mingled at 12 weeks of age [16]. Spatial analysis carried out in Denmark and Great Britain concluded the existence of significant spatiotemporal clusters, suggesting the spread of an infectious agent from farm to farm [17, 18]. Descriptive epidemiology in Sweden also showed a clear tendency of the epidemic to move slowly from south to north [19]. 

Previously we have shown that PMWS can be transmitted from pig to pig by close contact [20] and PCV2 has been found in air samples collected in PCV2-positive herds [21], but it still remained unclear if PMWS can be transmitted through air. The purpose of the present studies was to examine the possibility of airborne transmission of PMWS in a controlled semiexperimental setup.

## 2. Materials and Methods

### 2.1. Air Transmission Model

Two studies were performed. For the studies, three containers were constructed as “pig units” (unit A, unit B, and unit C). Units A and B were placed one meter apart and connected by pipes (Tables 2–4). In unit A, air pressure was increased by a ventilator mounted in the gable that blew fresh air into the room through four adjustable valves. Exhaust air was pushed out through a stack in the roof. In unit B, air pressure was decreased by a ventilator mounted in a stack in the roof that controlled exhaust air to the outside. Air was sucked into the room through four valves. Thus, air pressure in unit A was always higher than the air pressure in unit B, resulting in air transfer from unit A to unit B through the pipes. The volume of air transferred through the pipes depended on the number and diameter of the pipes as well as the pressure difference between the units. Thus, to maintain a particular rate of air transfer, the pipes diameter could be adjusted with orifices. The ventilation system, the opening valves, and the pipes were all calibrated before the beginning of the studies. Air pressure differences were measured every 10 min in order to calculate the amount of air transferred. The amount of air transferred from unit A to unit B, expressed as percent of ventilation intake, was on average 83% (S.D. 27%) for study I and 69% (S.D. 27%) for study II. 

Units A and B were 2.5 × 9.5 meter each and consisted of two rooms. Personnel entered through the first room (2.5 × 2.5 meter) and changed clothes there. The pigs were housed in the second room which was 17.5 m^2^ and had slatted plastic floor. Each unit had a two-climate system with coverings and straw bedding. Units A and B were placed at research facility 1, 2.2 km from other pig herds.

Unit C was placed at research facility 2 approximately 3.1 km from unit A and unit B, with no pigs within a range of 2.3 km from the unit. The unit was 18 m^2^ and consisted of one room with slatted plastic floor and a two-climate system with coverings and straw bedding. 

### 2.2. Study Setup

The pigs (Danish Landrace/Duroc crossbred), 8–12 weeks of age, were obtained from three different herds that were all PCV2 infected and PCV2 unvaccinated, verified by serological reactions. Two of the herds were PMWS affected (PMWS-1 and PMWS-2) and one was PMWS-free (PMWS-free). The PMWS diagnosis within the PMWS-affected herds were based on high prevalence of unthrifty pigs, high mortality among weaners (5-6% and 15%), and a positive histopathological examination of lymphoid tissue together with detection of PCV2 antigens according to the EU definition (http://www.pcvd.org/). The PMWS-free herd was characterized by low mortality among weaners (2-3%). This status persisted during the study period and until three months after. 

All three herds were infected with *Mycoplasma hyopneumoniae* and *Actinobacillus pleuropneumoniae* serotype 6. The PMWS-1 and the PMWS-free herds were infected with PRRSV-EU and PMWS-2 with *A. pleuropneumoniae* serotype 2, toxigenic *Pasteurella multocida*, and PRRSV-US. A vaccine against porcine parvovirus was used in sows in all three herds. A vaccine against toxigenic *P. multocida* was used in herd PMWS-1.

The PMWS-affected herds were visited by the veterinarian 3-4 days before the start of each of the studies at which time 25 (31 for study II) pigs with clinical symptoms of PMWS among weaners were selected and ear tagged. The same veterinarian visited the PMWS-free herd one week before the study and started to make sure that no clinical signs of PMWS were present. From the PMWS-free herd, 105 pigs were transferred to the research facilities at weaning in each study. 

The pigs from the PMWS-nonaffected herd were randomly assigned to unit A, unit B, and unit C. Approximately half of the pigs in unit A and all the pigs in unit B and C originated from the PMWS-free herd in both studies. The remaining pigs in unit A originated from the PMWS-affected herd PMWS-1 in study I and PMWS-2 in study II (Table 1). 

On the day of arrival (day 1), all the pigs were marked with individual ear tags and weighed. 

Water and feed without antibiotics were offered *ad libitum* throughout the study period. To prevent diarrhoea 2500 ppm zinc oxide was used in the feed the first 10 days after arrival in both studies.

Transmission of pathogens by personnel was prevented through biosecurity measures (changing clothes before entering units, using disposable gloves and masks covering hair, nose, and mouth). A twelve-hour pig contact quarantine was established before entering the units, within which a shower had to be taken and clothes changed. The pigs were handled in the same order every day: first unit C, then unit B, and finally unit A. Transmission by insects was prevented by a fly net at the air inlet in unit B. The duration of both studies was 69 days.

### 2.3. Clinical Observation and Postmortem Examination

The pigs were monitored for clinical signs of PMWS three times weekly by the veterinarian. 

All the pigs that demonstrated severe clinical disease were euthanized during the study. At the end of the study, all the unthrifty pigs were euthanized. All the pigs that were euthanized or died spontaneously were necropsied.

During necropsy, a tissue sample was taken from the inguinal lymph node, the mesenterical lymph nodes, and the spleen and fixed by immersion into 4% paraformaldehyde at 22°C for histopathological examination. Sections of paraffin-embedded paraformaldehyde-fixed tissue were stained with hematoxylin and eosin for histomorphological evaluation and immunohistochemistry for PCV2 by specific monoclonal antibodies as previously described [22]. The individual pigs were diagnosed with PMWS if they showed clinical signs together with characteristic histopathological lesions in lymphoid tissue (lymphocyte depletion together with histiocytic infiltration and/or giant cells and/or inclusion bodies) and detection of moderate or massive amounts of PCV2 antigen by immunofluorescence. This is in accordance with the EU definition (http://www.pcvd.org/).

### 2.4. Blood Sampling and Serological Analysis

Blood samples were collected from all the pigs at the beginning of the studies. Additional blood samples were collected from the pigs before euthanization or at termination of the study. Blood was rescued from the heart of dead pigs when possible.

The serum samples were tested for PRRSV antibodies using immunoperoxidase monolayer assay (IPMA) as previously described [23]. The IPMA was carried out as a double test [24] using MARC-145 cells infected with a Danish field strain of PRRSV and with an American vaccine strain (“Ingelvac” PRRS MLV, Boehringer Ingelheim), respectively. Antibodies against PCV-2 were measured by an in-house developed ELISA [20]. Tests for antibodies against PCV2, PRRS (Types I and II), and PCV2 quantification were only carried out on samples from study II.

The geometric mean was calculated for each group and sampling in study II as the exponential of the arithmetic mean of the log-transformed titer values (>0) (Table 4). The proportion of samples with a titer value above 0 was also determined (Table 4).

### 2.5. Real-Time PCR and Sequencing

In study II, PCV2 was quantified by real-time PCR on serum samples from selected pigs from the PMWS-affected herd in unit A and from all pigs in unit B at arrival and, when blood was rescued, at death or at termination of the study. Real-time PCR was performed as previously described [25]. The PCV2 genome of selected samples was PCR amplified in three overlapping reactions and sequenced as previously described [15, 26].

## 3. Results

### 3.1. Clinical Signs and Postmortem Examination

The mean weight of the pigs at arrival is listed in Table 1. In study I, one of the pigs in unit B died on day 2 after the beginning of the study due to diarrhea and one of the pigs was euthanized due to lameness four weeks after the beginning of the study. No other clinical signs were seen among the pigs from the PMWS-free herd in the three units. Fourteen of the pigs from the PMWS-affected herd in unit A were necropsied. Nine of these pigs were diagnosed with PMWS according to the EU definition (Table 2).

In study II, the pigs from the PMWS-free herd in unit A started to show clinical signs of PMWS two weeks after mingling with the pigs from the PMWS-affected herd. In unit B, receiving air from unit A, the clinical signs were seen three weeks after the start of the study. The most prominent clinical signs were depression, unthriftiness, and wasting. Some of the pigs had dyspnea or diarrhoea. At necropsy the predominant findings were enteritis followed by heavy lungs and bronchopneumonia. Many pigs had enlarged bronchial and inguinal lymph nodes and some had enlarged mesenteric lymph nodes (Table 2). Fourteen of the pigs from the PMWS-affected herds in unit A were necropsied, and three of these were diagnosed with PMWS. Ten of the pigs from the PMWS-free herd in unit A were necropsied, and three of these were diagnosed with PMWS (Table 3). Of the pigs in unit B, 20 were necropsied and 13 of these were diagnosed with PMWS. In both units approximately one-third of the pigs died or were euthanized due to evident wasting. None of the pigs in unit C showed any signs of wasting or any other clinical symptoms of disease. Eight of the healthy pigs from unit C were euthanized and necropsied and showed no signs of PMWS.

### 3.2. Serological Analysis

The blood samples from study I were not analyzed due to lack of clinical signs and confirmed PMWS diagnosis in pigs from the PMWS-free herd. 

The serological profiles of the different groups at arrival and at termination of study II are shown in Table 4. The pigs from both the PMWS-free and the PMWS-affected herds had antibodies against PCV2 at arrival. The levels of antibodies in the pigs from the PMWS-affected herd were significantly higher than the levels in the pigs from the PMWS-free herd. A marked increase in the level of PCV2 antibodies was seen in all the pigs in units A and B in contrast to a decrease in the level of PCV2 antibodies seen in the pigs in unit C at facility 2.

The pigs from the PMWS-affected herd had high levels of antibodies against PRRSV Type II (US) at arrival and the level remained high until the end of the study. The pigs from the PMWS-free herd had no antibodies against PRRSV Type II at arrival. Pigs from this herd placed in units A and B showed a marked increase in the level of antibodies towards PRRSV Type II from the start to the end of the study in contrast to pigs placed in unit C at facility 2 which did not develop antibodies against PRRSV (Table 4). Apart from the low level in some pigs, all pigs were free from antibodies to PRRSV Type I (EU) at arrival. 

### 3.3. PCV2 Levels in Serum and Sequencing

The blood samples from study I were not analyzed due to lack of clinical signs and confirmed PMWS diagnosis in pigs from the PMWS-free herd. 

In study II, from unit A 11 of the pigs from the PMWS-affected herd that were necropsied were tested by quantitative PCR for PCV2 at the start of the experiment. The three pigs that got a confirmed PMWS diagnosis had PCV2 titers of 1,5E + 10; 8,7E + 10; and 6,5E + 07, respectively, at the start of the experiment (data not shown). Blood was only rescued from one of these pigs at necropsy showing 4,0E + 11 copies of PCV2 pr. mL serum. The PMWS diagnosis could not be confirmed for the remaining eight pigs and these pigs had PCV2 titers at 7,3E + 07 copies of PCV2 pr. mL serum or lower at necropsy, which was slightly lower than at the start of the experiment.

**Figure 1:**
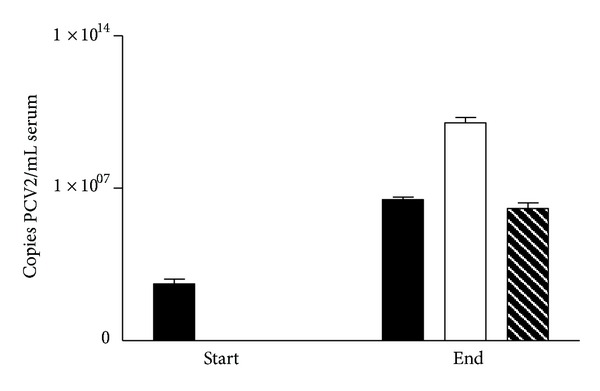
Level of PCV2 in serum (copies per mL of serum) of pigs from PMWS-free herds in unit B (study II) at arrival and at termination of the study. Pigs showing no signs of PMWS (black); pigs with confirmed PMWS (white) and pigs showing signs of PMWS, but which did not fulfil the criteria at necropsy (black and white).

From unit B, 38 pigs were tested for PCV2 DNA by quantitative PCR at arrival and at necropsy or at termination of the study (Figure 1). At the start of the experiment, PCV2 was either undetectable (35 pigs) or very low (three pigs). At the end of the experiment, they all developed high PCV2 titers ranging from 2,8E + 04 to 6,4E + 10 copies of PCV2 pr. mL. Eight of the pigs had more than 6,7E + 07 copies of PCV2 pr. mL serum at necropsy and they were all confirmed PMWS cases by histopathological examination. Additionally four of the pigs analyzed with quantitative PCR were examined histologically due to clinical signs of PMWS but did not fulfil the histopathological criteria for PMWS diagnosis and they had lower PCV2 titers in the sera (from 3,9E  +  04 to 4,1E  +  06 copies pr. mL) (Figure 1).

PCV2 rescued from serum of six pigs from the PMWS-affected herd in unit A at arrival and from tissue collected at necropsy from two of the pigs from the PMWS-free herd which developed PMWS in unit B was sequenced. All sequenced isolates belonged to the genotype 2b and were closely related to circulating Danish PCV2 isolates [26] (data not shown). The positions of the few base differences identified in PCV2 genomes isolated from the pigs from units A and B are shown in Table 5. Two variants of PCV2 were isolated from pigs from the PMWS-affected herd in unit A on arrival. One of these variants was also found in both of the pigs that developed PMWS in unit B. 

## 4. Discussion 

To our knowledge this is the first controlled study that documents that PMWS can be induced in pigs from PMWS-free herds exclusively by receiving air from a unit harbouring pigs with clinical PMWS. Previously, we have shown that PMWS can be induced in healthy pigs from PMWS-free herds by direct or indirect contact with pigs from PMWS-affected herds [20] and others have induced PMWS after mingling of pigs inoculated with PCV2 and naïve pigs under experimental conditions [13, 14, 27]. None of the pigs from PMWS-free herds developed clinical signs of PMWS after transportation alone. Therefore, it seems unlikely that stress due to transportation induced PMWS by itself. The fact that the pigs from the PMWS-free herds in units A and B but not in C had a marked increase in PCV2 titer in serum and an increase in antibodies against PCV2 that coincided with the subsequent development of clinical signs typical of PMWS further supported the view that the pigs developed PMWS. 

The PMWS diagnosis could not be confirmed by laboratory investigations in a proportion of the pigs with severe clinical signs of PMWS. This has previously been reported from field cases and probably represents end-stage pigs in which the virus level in tissues is low because of massive destruction of cells [7].

The pigs from the PMWS-free herd developed clinical signs of PMWS two weeks after arrival and mixing if they had direct contact with the PMWS-positive pigs (unit A) whereas the pigs in unit B which had no direct contact with the PMWS pigs developed clinical signs of PMWS three weeks after the start of the study. Previous studies with experimental PCV2 infection in naïve pigs have shown that the transmission of PCV2 is influenced by the contact structure and that direct contact between pigs was more efficient in transmission compared to indirect contact (10 cm distance) [13]. Furthermore, the same authors found that the mean disease generation time was 18.4 days following contact with a diseased pig. Accordingly, we have previously shown that disease was induced in PMWS-free pigs 3-4 weeks following mingling with pigs from herds with clinical signs of PMWS and that close contact was more efficient compared to indirect contact [20]. Thus, the finding that the pigs in the present study developed clinical signs of PMWS 2-3 weeks after arrival indicates that the disease generation times in PCV2-positive pigs are comparable to the situation in naïve pigs and that airborne transmission does not delay development of clinical signs. 

The viral DNA sequences found in two of the pigs that developed PMWS in unit B were identical and also identical to one of the isolates found in PMWS-affected pigs at arrival. It was not possible to sequence the PCV2 virus from the PMWS-free herd at arrival; however the sequencing results support that the PCV2 virus was transmitted from the PMWS-affected pigs to the pigs from the PMWS-free herds. This is in accordance with our finding in an earlier study on transmission of PMWS by direct and indirect contact [15].

The low levels of antibodies to PRRSV Type I (EU) in the pigs from the PRRSV Type II (US) positive herd probably reflect cross-reaction to PRRSV Type II and the low levels in the remaining pigs probably reflect residue of maternal antibodies. The increase in levels towards PRRSV Type I encountered in some of the pigs at termination of the study probably reflects cross-reaction to PRRSV Type II since these levels were only detected in pigs with very high levels of antibodies against PRRSV US. These results strongly indicate that pigs from the PMWS-free herd in units A and B were infected by PRRSV US during the study period and that the source was pigs from the PMWS-affected herd. PRRSV is a well-known infectious trigger of clinical PMWS and vice versa [28–32] and we have previously shown that PRRSV can be transmitted by air [33]. Thus, the finding that most of the pigs that developed PMWS had increased antibody titers against PRRSV Type II suggested that this virus contributed to the clinical manifestation seen in study II. The clinical signs were, however, more in accordance with typical findings in PMWS-affected pigs rather than what is typically seen in pigs acutely infected with PRRSV [29]. The pathological findings at necropsies indeed confirmed that the pigs had developed PMWS. In addition to the role of PRRSV, factors such as differences in PCV2 strain, the dose of PCV2 virus excreted by the “donor” pigs, or even transmission of other unidentified infectious agents from the PMWS-affected pigs to the PMWS-free pigs may have played a role. In conclusion, the present study showed that PMWS can be induced in pigs from a PMWS-free herd by airborne contact with pigs from a PMWS-affected herd. This finding may have implications on the way PMWS is handled in herds.

## Figures and Tables

**Table 1:** Distribution of pigs in the two experiments.

	Facility 1			Facility 2
	Unit A		Unit B	Unit C
Experiment 1				
Status herd of origin	PMWS-1	PMWS-free	PMWS free	PMWS free
Number of pigs	25	25	50	30
Mean weight (kg)	14.2	11.7	11.0	11.3

Experiment 2				
Status herd of origin	PMWS-2	PMWS free	PMWS free	PMWS free
Number of pigs	31	25	50	30
Mean weight (kg)	14.3	9.3	9.3	9.0

**Table 2:** Gross lesions detected on autopsied pigs from the four units in experiment 2.

Gross lesions	Unit A		Unit B	Unit C
	PMWS-2	PMWS-free	PMWS-free	PMWS-free
Number of pigs autopsied	14	10	20	8
Heavy lungs	5	4	15	0
Bronchopneumonia	5	3	8	0
Enteritis/dilated intestines	12	9	16	0
Enlarged mesenterical lnn.	5	4	4	0
Enlarged bronchial lnn.	9	6	10	0
Enlarged inguinal lnn.	6	6	11	0

**Table 3:** Number of pigs diagnosed with PMWS.

Status herd of origin	Unit A		Unit B	Unit C
	PMWS pos.	PMWS-free	PMWS-free	PMWS-free
Experiment 1				
Number euthanized	14	0	2^1^	0
PMWS positive	9	0	0	0

Experiment 2				
Number euthanized	14	10	20	8
PMWS positive	3	3	13	0

^1^One euthanized due to diarrhea, another due to lameness.

**Table 4:** Geometric mean of positive antibody titers (proportion of positive samples) against PRRSV EU, PRRSV US, and PCV2, at arrival and at the end of study II.

Facility	1			2
Unit	A		B	C
Status of herd	PMWS affected	PMWS-free	PMWS-free	PMWS-free
PRRS EU				
Arrival	79.2 (*P* = 7/31)	50.0 (*P* = 2/25)	50.0 (*P* = 9/50)	50.0 (*P* = 2/30)
End	135.4 (*P* = 21/26)	123.7 (*P* = 16/23)	146.2 (*P* = 27/44)	n.a.* (*P* = 0/30)

PRRS US				
Arrival	326.9 (*P* = 30/31)	n.a. (*P* = 0/25)*	n.a. (*P* = 0/50)*	n.a. (*P* = 0/30)*
End	385.6 (*P* = 26/26)	503.3 (*P* = 23/23)	539.0 (*P* = 44/44)	n.a. (*P* = 0/30)*

PCV2				
Arrival	10887.2 (*P* = 29/31)	159.3 (*P* = 25/25)	267.0 (*P* = 49/50)	906.0 (*P* = 30/30)
End	177720.6 (*P* = 25/26)	54697.9 (*P* = 23/23)	13704.7 (*P* = 41/44)	31.9 (*P* = 25/30)

*n.a.: not applicable, because all titres were 0.

**Table 5:** Position of base differences identified in the full PCV2 genomes isolated from pigs from containers A and B.

Base position	350	1399	1405	1528
Pig 5 container A	G	G	C	C
Pig 31 container A	G	G	C	C
Pig 2 container A	A	T	T	T
Pig 9 container A	A	T	T	T
Pig 18 container A	A	T	T	T
Pig 28 container A	A	T	T	T
Pig 206 container B	A	T	T	T
Pig 217 container B	A	T	T	T
